# Enhancing 10-HDA production of *Escherichia coli* by heterologous expression of MexHID transporter proteins

**DOI:** 10.3389/fbioe.2025.1590291

**Published:** 2025-06-09

**Authors:** Ziting Xu, Chaofan Du, Sheng Gao, Xinrui Yan, Pan Deng, Yuehan Liu, Junqing Wang, Ruiming Wang, Nan Li

**Affiliations:** ^1^ School of Bioengineering, Qilu University of Technology, Jinan, Shandong, China; ^2^ State Key Laboratory of Green Papermaking and Resource Recycling, Qilu University of Technology, Jinan, Shandong, China

**Keywords:** transporter proteins, chromosome integration, metabolic engineering, whole-cell catalysis, medium-chain fatty acid

## Abstract

10-Hydroxy-2-decenoic acid (10-HDA) is a medium-chain α,β-unsaturated carboxylic acid that exists in royal jelly with terminal hydroxylation. It has a broad market value because of its antibacterial, anti-inflammatory, anti-tumor, anti-radiation, and other active functions. The one-step whole-cell catalytic synthesis of 10-HDA by constructing engineered strains has improved the reaction rate to a certain extent compared with the previous two-step method. However, the accumulation of 10-HDA to a certain concentration in engineered *Escherichia coli* strains will damage the structure and function of cells and even lead to death; this unique antibacterial and antimicrobial activity seriously constrains the production of 10-HDA. In this study, we mined a transporter protein from *Pseudomonas aeruginosa*, which possesses the ability to efficiently efflux 10-HDA, and constructed a transporter protein overexpression strain by using the multicopy chromosome integration technique, which further improved the efficiency of product efflux, weakened the feedback inhibition of 10-HDA to a certain degree, and increased the substrate conversion rate to 88.6%. 10-HDA was synthesized up to 0.94 g/L by the replenishment flow-addition technique, providing a simple and efficient pathway for the yield breakthrough of 10-HDA biosynthesis.

## 1 Introduction

Royal Jelly (RJ) is a liquid prepared from the milky white gel secreted by the pharyngeal glands of worker bees and honey, with high nutritional value. 10-HDA, as the main substance that plays an active role in RJ, has anti-bacterial (Oršolić and Jazvinšćak Jembrek, 2024), anti-inflammation (Yang et al., 2018; Bagameri et al., 2023), radiation protection (Mirbaha et al., 2019; Huang et al., 2024), and anti-cancer properties (Filipič et al., 2015; Lin et al., 2020). Among the existing methods for obtaining 10-HDA, traditional physical extraction and chemical synthesis have many drawbacks. For example, the raw materials are difficult to obtain, many by-products are produced, purification is difficult, the recovery rate is low, and operation is complicated. Therefore, biological methods have become mainstream owing to their environmentally friendly, nonpolluting nature, and high production efficiency; however, there is a serious feedback inhibition phenomenon of the substrate and product, which limits the maximum yield of 10-HDA to 0.628 g/L (Fang et al., 2024).

Transporter proteins play a key role in intra- and extra-cellular substance exchange and can be carrier or channel proteins responsible for mediating active and passive transport (Cocucci et al., 2017; Varela et al., 2023). The resistance-nodulation-division (RND) family of transporter proteins, which are widely distributed in Gram-negative bacteria, catalyze intracellular reactive substances (e.g., bile acid salts and fatty acids) and the exocytosis of metal ions (Cu^+^, Ag^+^) (Delmar et al., 2015; Venter et al., 2015; Henríquez et al., 2020). By mining and modifying specific transporter proteins, the efficiency of uptake and efflux of specific substrates or products by strains can be improved, and this is expected to be an effective means to relieve feedback inhibition and realize product accumulation (Abumrad, 1999; Colclough et al., 2020; Colclough et al., 2020; Ahmed et al., 2021). In Gram-negative bacteria, the RND superfamily efflux pumps play a crucial role in antibiotic resistance, and one of the ways in which multidrug resistance can be generated in *Escherichia coli* and *Pseudomonas aeruginosa* is through RND efflux pumps (Zahedi bialvaei et al., 2021). Marine’s study revealed that the mechanism of adaptive antibiotic resistance in Gram-negative bacteria stems mainly from the action of the RND family of transport proteins (Novelli and Bolla, 2024). The timely excretion of 10-HDA from intracellular to extracellular using transporter proteins can increase the secretion of the product, thus enhancing its accumulation in the fermentation broth and increasing the yield. High levels of butanol (>14 g/L) were produced by overexpressing srpB, an efflux pump gene from *Pseudomonas* putida, to enhance the tolerance of high butanol-producing clostridia to fermentation inhibitors in a study by Pablo et al. (Jiménez-Bonilla et al., 2020).

Optimizing the dosage of specific genes for their efficient expression is a critical step in engineering high-performance biocatalysts (Jodlbauer et al., 2024). Plasmid expression systems and multicopy chromosome integration are two commonly used gene expression strategies in microbial engineering. Plasmid expression systems are fast to construct and flexible to operate, but their metabolic processes consume a large amount of host resources, resulting in reduced growth rates and loss of plasmids in long-term passages, making them genetically unstable, and precise control of the dosage of gene expression cassettes is challenging due to the limited number of plasmid replicons and the variety of external factors affecting the plasmid copy number. The CRISPR/Cas system, as part of multiple types of adaptive immune systems involving multiple host DNA repair pathways, is a more precise and efficient genome editing tool that requires fewer components (Klompe et al., 2022). Multicopy Chromosome Integration Technology Using CRISPR-associated Transposons for Chromosome Integration (MUCICAT) can be implemented by designing crRNAs to target multiple replication sites in the *E. coli* genome or crRNA arrays to target multiple sites in the *E. coli* genome, and this method of chromosome integration provides precise gene dosage control and recombination genetic stability (Gu et al., 2015; Zhang et al., 2021). Hou et al. in their study of L-tryptophan integrated an optimized expression cassette into the genome of *E. coli* by using a CRISPR-associated transposase system and balanced cell growth and product synthesis by adjusting the copy number of the expression cassette to achieve a final yield of 5.1 g/L of L-tryptophan in shake flask fermentation (Hou et al., 2025).

In our previous study, we used engineered *E. coli* expressing Macs, FadE, YdiI, CYP, and ACOX to catalyze the synthesis of 10-HDA from decanoic acid in a one-step process (Fang et al., 2024), which improved the conversion rate and yield compared to the two-step process (Li et al., 2022), but feedback inhibition due to the product still existed. To address this issue in this study, we identified *P. aeruginosa* (*P. aeruginosa*) capable of growing under higher concentrations of 10-HDA stress through strain screening. Comparison of 10-HDA tolerance and transport capacity was performed by genome sequencing and annotation searching for transporter proteins, and the function of the transporter protein MexHID was determined. We validated and compared the expression of transporter proteins using both plasmid expression systems and multicopy chromosome integration. Transportation of 10-HDA from intracellular to extracellular space via transporter proteins was an effective strategy, decreasing the concentration of intracellular 10-HDA and reducing its toxicity to cells while increasing the substrate conversion rate and yield. In addition, this method simplifies the downstream purification process because 10-HDA is translocated from intracellular to extracellular space in the fermentation broth. Thus, 10-HDA can be extracted directly from the fermentation broth, which not only improves the synthesis efficiency but also makes the production of 10-HDA more convenient and economical.

The results of this study not only provide new ideas for the biosynthesis of 10-HDA but also provide an important reference for the cell factory production of other bioactive molecules. By optimizing the expression and function of transporter proteins, we can further improve the yield and cell tolerance of target products and open up new opportunities for the application of biotechnology in the medical, food, and chemical industries.

## 2 Materials and methods

### 2.1 Strain and medium

*E. coli* BL21 and *P. aeruginosa* were cultured in LuriaBertani (LB) liquid medium at 37°C and 200 rpm. Recombinant *E. coli* was inoculated in LB liquid medium containing ampicillin (100 μg/mL), streptomycin (50 μg/mL), and kanamycin (50 μg/mL) at 37°C and 200 rpm and screened at 37°C using LB solid medium (containing 2% agar). Decanoic acid or 10-HDA (Macklin, Shanghai, China) was used as the substrate during the reaction and dissolved in dimethyl sulfoxide. All strains and plasmids used in this study are listed in Table1, while the primers used are listed in Table2.

**TABLE 1:** Strains and plasmids used in this study.

Strain/plasmid	Relevant genotype/description	Source
pET28-*ydiI*	the expression vector of *E. coli* BL21, for expression of *ydiI* gene	in our previous research
pRSFDuet-1-*ydiI*	the expression vector of *E. coli* BL21, for expression of *ydiI* gene	this study
pETDuet-1-Parg-*acox*-T7-*cyp*	the expression vector of *E. coli* BL21, for expression of *cyp* and *acox* genes	in our previous research
pRSFDuet-1-*macs*- *fadE*	the expression vector of *E. coli* BL21, for expression of macs and *fadE* genes	in our previous research
pRSFDuet-1-*ydiI*-*mexHID*	the expression vector of *E. coli* BL21, for expression of *ydiI* and *mexHID* genes	this study
*E. coli*-ΔBRJ	*E. coli* BL21 strain knockout of *fadR*, *fadB* and *fadJ* genes	in our previous research
*E. coli* BL21 (DE3)	ompT hsdS (rB-mB-) gal dcm (DE3)	Vazyme
*P. aeruginosa*	Screening of 10-HDA tolerant strains identified in samples	this study
pRSFDuet-1-*mdtAB/mdtC/acrEF/mdtEF/mexCDJ/mexHID/mexABM/mexEFN*	the expression vector of *E. coli* BL21, for expression of *mdtAB/mdtC/acrEF/mdtEF/mexCDJ/mexHID/mexABM/mexEFN* genes	this study
*E. coli*-AKS- *mdtAB/mdtC/acrEF/mdtEF/mexCDJ/mexHID/mexABM/mexEFN*(plasmid expression)	*E. coli* BL21 expressing *mdtAB/mdtC/acrEF/mdtEF/mexCDJ/mexHID/mexABM/mexE*F*N* and *macs, fadE, ydiI, acox*, and *cyp* genes as plasmids and knocking out *fadR*, *fadB*, and *fadJ* genes	this study
*E. coli-*AKS	*E. coli* BL21 expressing *macs, fadE, ydiI, acox*, and *cyp* genes and knocking out *fadR*, *fadB*, and *fadJ* genes	in our previous research
*E. coli-*ΔBRJ -*mexHID*-TRF1-7	Generation 1–7 strains of *E. coli* BL21 incorporating the *mexHID* gene and knocking out *fadR*, *fadB*, and *fadJ*	this study
*E. coli*-AKS-*mexHID-1-5*	*E. coli* BL21 expressed *macs*, *fadE*, *ydiI*, *acox*, *cyp* integrated *mexHID* proteins into the genome and knocked out *fadR*, *fadB* and *fadJ* genes	this study
pDonor	Vectors containing transposons carrying the cargo gene	GenScript
pDonor-*mexHID*	Donor expression vector with the Cargo region replaced with a fragment of the mexHID gene	this study
pInsert	Expression vector containing the transposable element tnsABC induced by IPTG	GenScript
pTarget	Expression vector containing CRISPR nuclease induced by IPTG and crRNA targeting the IS1 locus	GenScript
pCas	Expression vectors containing Cas9, SacB and sgRNA targeting the AmpR promoter induced by rhamnose	GenScript

**TABLE 2:** Primers used in this study.

Primers	Sequence (5′-3′)
Donor-F	GAT​CCG​GCT​GCT​AAC​AAA​GC
Donor-R	CCT​ATA​GTG​AGT​CGT​ATT​AAT​TTC​GCG
YdiI-F	tca​cca​cag​cca​gga​tcc​ATG​TCG​GAC​TCA​GAA​GTC​AAT​CAA
YdiI-R	gca​tta​tgc​ggc​cgc​aag​ctt​TTA​CAC​AAC​GGC​GGT​GGT​CA
matAB-F	tta​ata​cga​ctc​act​ata​ggA​TGA​AAG​GCA​GTT​ATA​AAT​CCC​GT
matAB-R	gct​ttg​tta​gca​gcc​gga​tcT​TAC​GCC​TCC​TCT​TCA​TGA​CG
mdtC-F	tta​ata​cga​ctc​act​ata​ggA​TGA​AGT​TTT​TTG​CCC​TCT​TCA​TT
mdtC-R	gct​ttg​tta​gca​gcc​gga​tcT​TAT​TGC​GCG​CTC​CTT​TTT​C
acrEF-F	tta​ata​cga​ctc​act​ata​ggA​TGA​CGA​AAC​ATG​CCA​GGT​TTT
acrEF-R	gct​ttg​tta​gca​gcc​gga​tcT​TAT​CCT​TTA​AAG​CAA​CGG​CG
mdtEF-F	tta​ata​cga​ctc​act​ata​ggA​TGA​ACA​GAA​GAA​GAA​AGC​TGT​TAA​TAC​C
mdtEF-R	gct​ttg​tta​gca​gcc​gga​tcT​TAC​GCT​TTT​TTA​AAG​CGG​GC
mexCDJ-F	tta​ata​cga​ctc​act​ata​ggA​TGG​TGT​TGC​CGG​TGG​AGG
mexCDJ-R	gct​ttg​tta​gca​gcc​gga​tcT​TAA​CTC​CTG​CCG​CCT​CGA
mexHID-F	tta​ata​cga​ctc​act​ata​ggA​TGC​AGA​AAC​CCG​TCC​TGA​TC
mexHID-R	gct​ttg​tta​gca​gcc​gga​tcT​TAA​CGG​TTG​GCC​CCG​GC
mexABM-F	tta​ata​cga​ctc​act​ata​ggA​TGC​AAC​GAA​CGC​CAG​CC
mexABM-R	gct​ttg​tta​gca​gcc​gga​tcT​TAG​GCC​TGG​GGA​TCT​TCC​T
mexEFN-F	tta​ata​cga​ctc​act​ata​ggA​TGG​AAC​AGT​CAT​CCC​ACT​TCT​CC
mexEFN-R	gct​ttg​tta​gca​gcc​gga​tcT​TAG​GCG​CTG​GGT​TGC​CA
qMexHID-	ATG​CAG​AAA​CCC​GTC​CTG​ATC​GCC​AGT​GCC
qMexHID-R	TGC​CGG​CGA​AAC​CGC​CGG​GGC​CAA​CCG​TTG​A

### 2.2 Strain screening and characterization

Samples originating from the soil, cellar mud, and dacron were collected by adding 0.9% NaCl solution and 2–3 glass beads, shaking well, and centrifuging at 5,000 rpm for 10 min to collect the supernatant. The mixed bacterial solutions were inoculated into an LB medium containing 0.5, 1, or 2 g/L 10-HDA and incubated for 24 h to observe the growth. Cultures showing bacterial growth were selected as templates for 16S rRNA sequencing to obtain information on the 10-HDA-tolerant strains.

### 2.3 Selection of transporter proteins

Based on the results of the genome annotation of the 10-HDA-tolerant strain *P. aeruginosa*, four different transporter proteins—MexCDJ, MexHID, MexABM, and MexEFN—were selected. Moreover, based on previous reports and homologous comparisons, the transporter proteins MdtAB, MdtC, AcrEF, and MdtEF, which are derived from *E. coli*, were chosen to test product Tolerance and Product Discharge Capability. The genomes of *E. coli* and *P. aeruginosa* were extracted as templates for polymerase chain reaction (PCR), amplified using primers specific for MatAB, MdtC, AcrEF, MdtEF, MexCDJ, MexHID, MexABM, and MexEFN, and gel purified to obtain the corresponding gene fragments. The pET28a-*ydiI* plasmid was used as a template, and PCR reactions with YdiI-F and YdiI-R were performed to amplify YdiI and obtain a gene fragment of 714 bp in size by gel purification. Then, the pRSFDuet-1 plasmid was linearized using the ClonExpress II OneStep Cloning Kit, the cloning site 1 (MCS1) between ligated YdiI was selected, and the recombinant plasmid pRSFDuet-1-*ydiI* was finally obtained. The *Eco*RⅤ and *Kpn*Ⅰ restriction endonucleases of MCS2 were selected to linearize the plasmid pRSFDuet-1-*ydiI* by double digestion, and the recombinant plasmid was obtained by integrating the gene fragment of the transporter protein into pRSFDuet-1-*ydiI* vector by using the seamless cloning technique pRSFDuet-1 -*ydiI-*(*mdtAB/mdtC/acrEF/mdtEF/mexCDJ/mexHID/mexABM/mexEFN)* The recombinant plasmid pRSFDuet-1 -*ydiI*-(*mdtAB/mdtC/acrEF/mdtEF/mexCDJ/mexHID/mexABM/mexEFN*) was combined with the preconstructed plasmid pETDuet-1-Parg-*acox*-T7-*cyp*, and pCDFDuet-1-*macs*-*fadE* was transformed into *E. coli*-ΔBRJ to obtain recombinant *E. coli-*AKS-(*mdtAB/mdtC/acrEF/mdtEF/mexCDJ/mexHID/mexABM/mexEFN*) (plasmid expression). Positive clones were selected for the total synthesis of 10-HDA after incubation on LB agar plates containing a final concentration of 100 μg/mL Amp, 50 μg/mL Kan, and 50 μg/mL Str, with growth at 37°C for 16 h.

LB medium with 0.5, 1, 1.5, and 2 g/L 10-HDA was added, and the activated *E. coli-*AKS-X (plasmid expression) strains were inoculated into the 10-HDA medium with different concentrations of 10-HDA at 1% inoculum, while final concentration of 100 μg/mL Amp, 50 μg/mL Kan, and 50 μg/mL Str was added to give a certain screening pressure. Then, 0.5 mM isopropyl β-D-thiogalactoside (IPTG) was added to induce the expression of transporter protein. The strains were incubated at 37°C and 200 rpm. The OD_600_ of the strains was measured at 3, 6, 9, 12, and 24 h of incubation, and the growth was recorded.

### 2.4 Induced transposition of engineered strains

The pDonor plasmid was linearized and ligated to the *mexHID* gene fragment using the ClonExpress II OneStep Cloning Kit to obtain the recombinant plasmid pDonor-*mexHID*. Several colonies were scraped off from the plates after transformation of pDonor-*mexHID*, pInsert, and pTarget into *E. coli*-ΔBRJ, which were transferred to 50 mL of medium, to which Amp at a final concentration of 100 μg/mL, Kan at 50 μg/mL, and Str at 50 μg/mL were added to induce proteins related to transposition to be expressed. They were then placed in a shaker at 37°C and incubated at 200 rpm for 24 h. A portion of the culture solution was transferred to 50 mL of culture medium at an inoculum volume of 1%, to which Amp at a final concentration of 100 μg/mL, 50 μg/mL of Kan, 50 μg/mL of Str, and 1 mM of IPTG were added. Then, they were placed in a shaker at 37°C and incubated at 200 rpm for 16 h of incubation.

The strain at the end of 1 mM IPTG induction was called first generation bacteria. To improve the integration rate of the strain, a portion of the culture medium of the first generation strain was transferred to 50 mL of culture medium containing Amp at a final concentration of 100 μg/mL, Kan at 50 μg/mL, Str at 50 μg/mL, and IPTG at a final concentration of 0.1 mM was added for the induction of transposition. Finally, the culture solution was placed in a shaker at 37°C and incubated at 200 rpm. After 16 h of induction incubation, the second generation of bacteria, and a total of seven transfers were produced by continuous culture according to this method. The first–seventh generations of bacteria with genome copies of the *mexHID* gene were obtained at the end of the transposition.

### 2.5 Plasmid elimination of engineered strains

The 1-7 generation passaged strain of *E. coli*-ΔBRJ -*mexHID* was named *E. coli-*ΔBRJ -*mexHID*-TRF1-7. *E. coli*-AKS-*mexHID*-TRF1-7 were prepared as chemoreceptor cells. The pCas plasmid was transformed into *E. coli-*AKS*-mexHID-*TRF*-*1-7. The recombinant strains were cultured on LB agar plates containing 50 μg/mL Ampicillin and 10 mM rhamnose, and after 16 h of growth at 37°C, colonies growing on the outside were selected and inoculated onto four different LB solid plates containing 100 μg/mL Amp, 50 μg/mL Kan, 50 μg/mL Str, and 10 g/L sucrose. The inoculated plates were sealed and transferred to a thermostatic incubator and incubated at 37°C. Strains that could not be grown on plates containing Amp, Kan, and Str and that grew on sucrose plates eliminated the pDonor, pInsert, and pTarget plasmids. The plasmid elimination efficiency of the strains was calculated by counting the growth of the strains on the plates, *E. coli*-ΔBRJ -*mexHID-*1-7 is a generation 1-7 strain that integrates the *mexHID* gene and eliminates the pDonor, pInsert, pTarget plasmids.

### 2.6 Detection of gene integration number in engineered strains

After inducing expression of the *E. coli-AKS-mexHID-*1-7 strain, total RNA was extracted using the Total RNA Extraction Kit. qMexHID-F and qMexHID-R were designed as specific primers for qPCR based on the first 500 bp of the MexHID base sequence. qMexHID-F and qMexHID-R were used to set up the total quantitative PCR using GapA from *E. coli* as an internal reference, and a fluorescence quantitative PCR instrument was used to detect mRNA abundance. The thermal cycling parameters of the reaction were 95°C for 2 min, 95°C for 10 s for 40 cycles, 59°C for 20 s, and 72°C for 20 s. The final lysis curves were obtained at 59°C–95°C in increments of 0.5°C/5 s. The number of cargoes copied to their genome was determined by measuring the relative mRNA abundance of MexHID in the first–seventh generation strains. All mRNA relative abundances in the samples were calculated by the 2^−ΔΔC^_T_ method, and three independent biological replicates were used for all measurements.

The genome of *E. coli*-AKS-*mexHID*-5 was extracted as a template according to the specific requirements of Vazyme’s DC103 kit, and primers were designed according to the 28 IS1 loci for polymerase chain reaction (PCR).

### 2.7 Control of conditions for the synthesis of 10-HDA by recombinant strains

Remove individual colonies from the plate, resuspended in 3 mL LB medium, and incubated overnight. The culture was inoculated with 1% inoculum into 200 mL TB medium containing the corresponding resistance and incubated until the OD_600_ of the bacterial solution reached approximately 0.8–1.2. Hydrochloride, 5-ALA, and 0.5 mM FeCl_3_ were then added at 20°C and 200 rpm for 30 min, and then 0.5 mM IPTG was added to continue the induction for 20 h. The fermentation broth was centrifuged at 5,000 rpm for 10 min, and after the culture broth was removed, the cells were washed with PBS (pH 7.4) two or three times. Finally, the cells were suspended in 20 mL of the supplemented medium. Cells were suspended in 20 mL of M9 medium supplemented with 1% glycerol, 0.4% glucose, and appropriate antibiotics and whole-cell catalyzed at 30°C after the addition of decanoic acid as substrate. One-milliliter samples were collected at 2, 4, 6, 8, and 10 h after the start of the reaction, and the products were detected after sample processing using GC-MS.

### 2.8 Sample pretreatment and assay methods

The samples were pretreated as described in our previous paper. The sample composition was analyzed using GC-MS (Agilent 5977 A, Palo Alto, CA). An HP-5 MS capillary column was used with high-purity helium (99.9%) as the carrier gas and a constant-flow injection mode with an injection volume of 1 μL, split ratio of 1:50, and injection temperature of 250°C. The flow rate was maintained at 1 mL/min, started at a constant temperature of 50°C for 1 min, and then increased to 250°C at 15°C/min; the entire process lasted 10 min. The mass spectrometry conditions were 70 eV electron bombardment with ionizing electron energy, a doubling voltage of 1.89 kV, quadrupole and ion source temperatures of 150°C and 230°C, respectively, and scanning range controlled from 40 to 500 amu.

### 2.9 Batch replenishment fermentation

Flow-addition culture is a semicontinuous culture in which fresh medium is replenished intermittently or continuously to maintain high-density cell growth and increase the yield of target products. During flow addition, the reaction temperature is maintained at 37°C to ensure enzyme activity and a smooth reaction. We used 0.7 g/L decanoic acid as the initial substrate concentration because the conversion rate was maximized at this concentration. Flow-addition experiments were conducted starting at the 12th hour of completion of the reaction by adding 0.1 g/L every 3 h and continuing until the final concentration of 1.3 g/L, when the reaction was completed.

### 2.10 Statistical analysis

Statistical analyses were performed using the IBM SPSS Statistics version 29. One-way ANOVA was used to analyze the significance of the mean differences between the samples. Significant differences were estimated at a 0.05 confidence level.

## 3 Results and discussion

### 3.1 Screening and identification of 10-HDA tolerant strains and selection of efficient transport proteins

**FIGURE 1:**
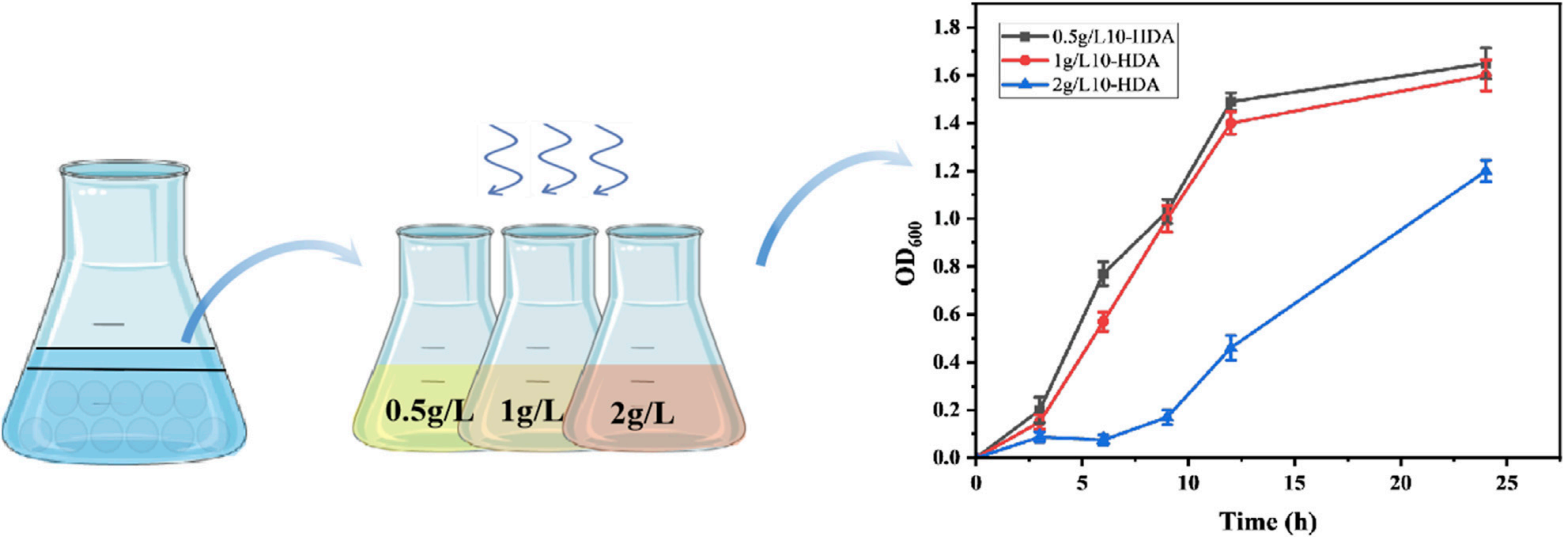
Samples were treated with glass bead shock, sorted and cultured in different 10-HDA stress environments to screen for 10-HDA tolerant strains as well as growth rates of the screened strains at different 10-HDA stress concentrations. Error bars represent standard deviations.

Using samples from different sources as libraries for screening 10-HDA-tolerant bacteria, it was observed that the soil-derived microbial population contained potential 10-HDA tolerance (Figure 1), which was successfully identified as *P. aeruginosa* by sequencing. A study by Chevalier et al. indicated that *P. aeruginosa* has high intrinsic resistance, and our study corroborated the same (Chevalier et al., 2017). It is hypothesized that *P. aeruginosa* transports harmful substances (10-HDA) to the extracellular compartment through the role of transporter proteins to minimize cellular damage; The ability of transporter proteins from *E. coli* to transport fatty acids was initially reported in a previous study by Wang et al. (Wang et al., 2022). In combination with Wu Junjun et al. who had previously shown that expression of the drug-resistant nodule cell division family transporter proteins AcrE, MdtE, and MdtC resulted in an increase in MCFA titer of 46.4% (Wu et al., 2019). Therefore, the transporter proteins MdtAB, MdtC, AcrEF, MdtEF derived from *E. coli* and the transporter proteins MexABM, MexCDJ, MexEFN, and MexHID from *P aeruginosa* were selected for testing.

**FIGURE 2:**
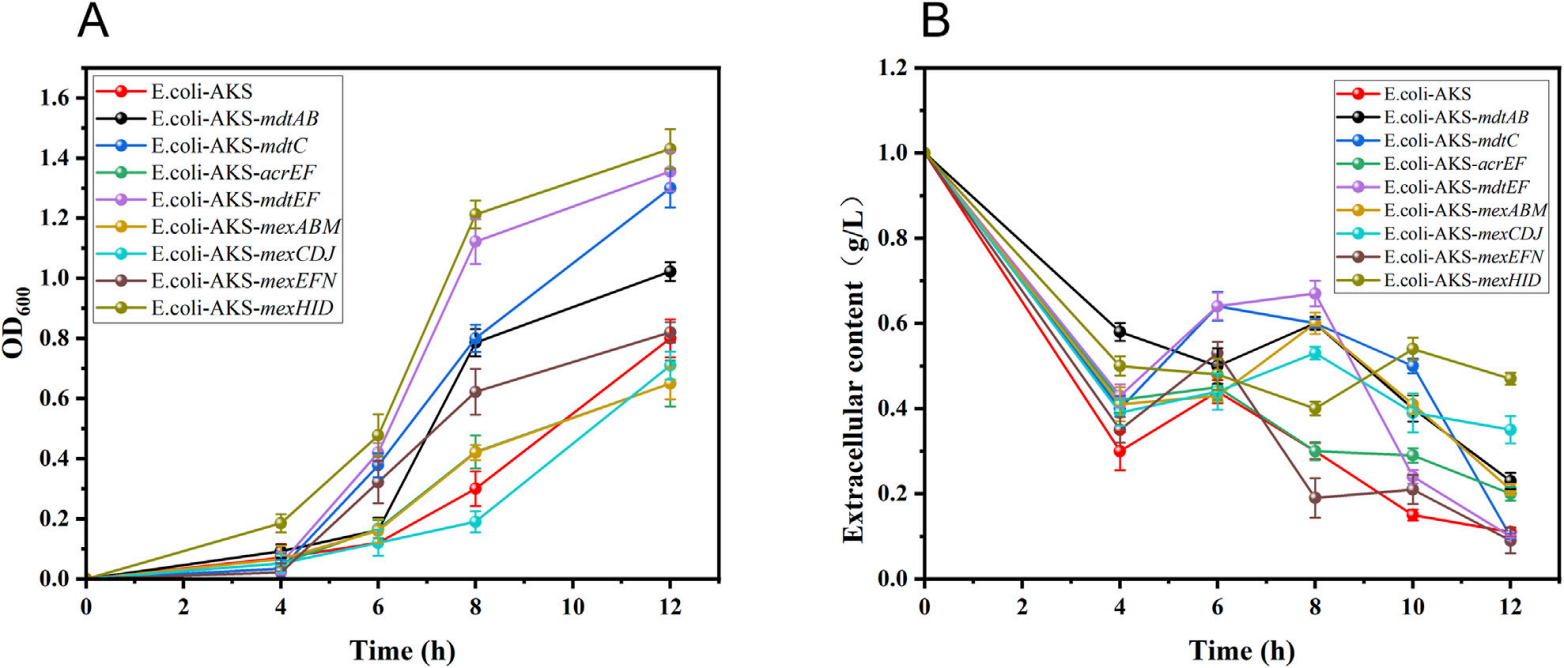
**(A)** Growth rates of different transporter protein-expressing strains under 10-HDA stress at 1 g/L concentration. Error bars represent standard deviations. **(B)** Changes in extracellular 10-HDA concentration in different transporter protein-expressing strains under 1 g/L 10-HDA stress. Error bars represent standard deviations.

They were constructed into the engineered strain *E. coli*-AKS, which had previously synthesized 10-HDA in a one-step process, for the validation of tolerance and translocation capacity, respectively. *E. coli*-AKS contains plasmids pETDuet-1-Parg-*acox*-T7-*cyp*, pCDFDuet-1-*macs*-*fadE*, and pET28a -*ydiI*. In order to avoid the plasmid stability problem and gene expression interference caused by the incompatibility of the replication start point of the ColE1-type, the vector of *ydiI* gene was replaced from pET28a to pRSFDuet-1 and eight different transporter proteins were constructed into another site of pRSFDuet-1 by restriction enzyme digestion and homologous recombination technology. The vector of *ydiI* gene was changed from pET28a to pRSFDuet-1 by restriction enzyme digestion and homologous recombination, and eight different transporter proteins were constructed into the other site of pRSFDuet-1, and the process of plasmid construction is shown in Supplementary Figure S1. The recombinant plasmids pRSFDuet-1-*ydiI*-(*mdtAB, mdtC, acrEF, mdtEF, mexCDJ, mexHID, mexABM, and mexEFN*) were combined with the pCDFDuet-1-*macs*-*fadE* plasmid, as well as the pETDuet-1-Parg-*acox*-T7-*cyp* plasmid, by transformation into the *E. coli*-ΔBRJ. Eight recombinant strains of *E. coli*-AKS-X (plasmid expression) were finally obtained. The tolerance (i.e., growth) of each strain when fed different concentrations of 10-HDA (Supplementary Figure S2). The highest concentration of 1 g/L, at which the strains could grow normally, was used as the stress concentration according to the growth status of the strains at different concentrations of 10-HDA, and the growth and extracellular 10-HDA content at different times were used for the comparison of the transporter capacity. In the case of 1 g/L 10-HDA stress, it was found that the strains underwent different levels of growth, where *E. coli*-AKS-*mexHID* (plasmid expression) had the fastest growth rate OD_600_ value of approximately 1.5, and was the first to reach the exponential phase of growth (Figure 2A). In combination with the measurement of the extracellular 10-HDA content in the case of 10-HDA stress (combined with the measurement of extracellular 10-HDA content under 10-HDA stress (1 g/L)). *E. coli*-AKS-*mexHID* (plasmid expressed) had the highest extracellular content of 0.47 g/L (Figure 2B), which was 5-fold higher than that of the control strain *E. coli*-AKS. This phenomenon indicated that the transporter protein could efficiently complete the process of product (10-HDA) exocytosis and became the most promising factor to overcome the bottleneck of the 10-HDA yield limitation.

### 3.2 Construction of transporter protein overexpression strains by multi-copy chromosome integration technology

Many naturally occurring multicopy sequences are present in the *E. coli* genome, such as the insertion sequence (IS). By designing crRNA targeting the IS site in *the E. coli* genome, the cargo gene on the donor plasmid can be targeted to the target integration site. Genome integration of the strain can be realized under the action of transposase, and the number of genes integrated into the genome of the strain can be increased through successive induced passages (Ou et al., 2018; Zhang et al., 2020). After genomic integration, the MexHID gene was introduced into multiple IS sites in the bacterial genome, and the number of integrated MexHID genes in the strain was increased with successive passages, resulting in a strain with a high copy number.

**FIGURE 3:**
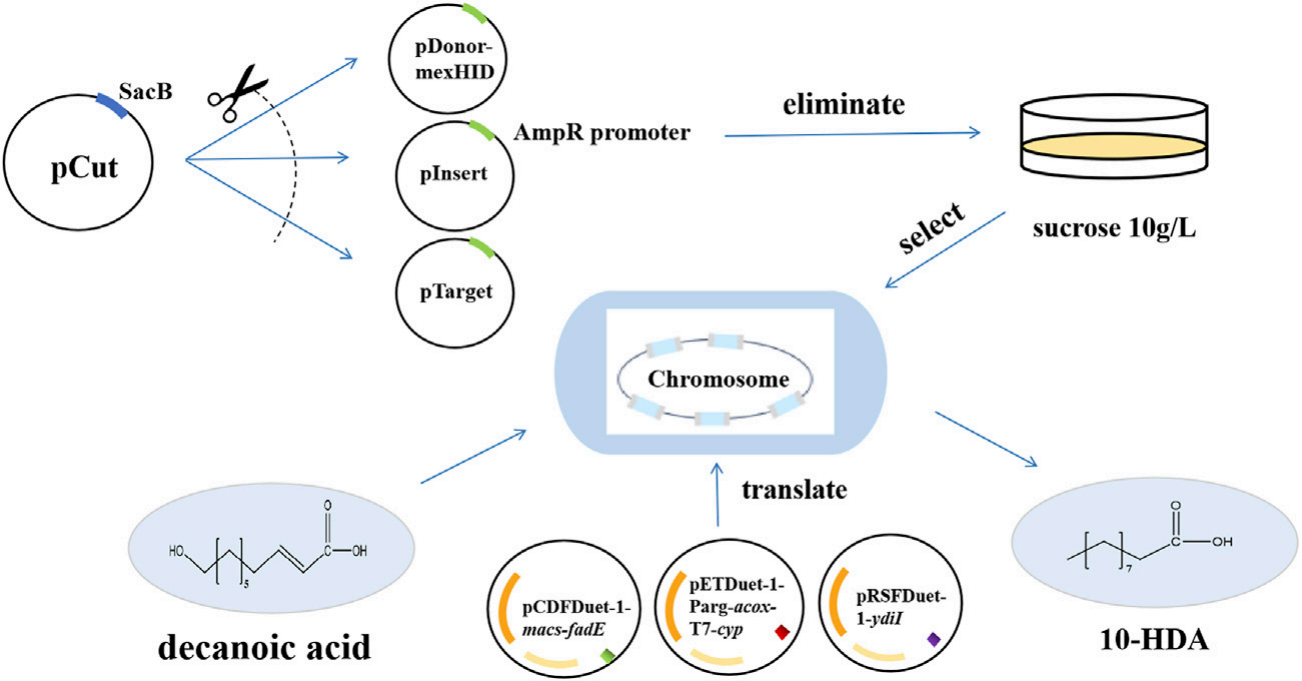
Technology roadmap for integrating the *mexHID* gene, assembling the biosynthetic 10-HDA catalytic element and performing 10-HDA total synthesis using multicopy genome integration technology.

The pCas plasmid contained sgRNAs that simultaneously target the Ampr promoter in the pDonor, pInsert, and pTarget plasmids, as well as Cas9 derived from *Streptococcus pyogenes*, which was transformed to cleave pDonor, pInsert, and pTarget induced by rhamnose to achieve plasmid elimination. The pCas plasmid was used to cleave the pDonor, pInsert, and pTarget plasmids. The pCas plasmid also contains SacB (Qin et al., 2021; Zhou et al., 2024), a protein whose sucrose component in the medium leads to strain virulence after expression; therefore, *E. coli* carrying pCas cannot grow in plates containing 10 g/L sucrose, the pCDFDuet-1-*macs-fadE*, pETDuet-1-Parg-*acox*-T7*-cyp*, and pRSFDuet-1-*ydiI* were transfected into plasmid-eliminated strains with mexHID genes to construct an engineered strain for 10-HDA synthesis (Figure 3). As shown in Supplementary Figure S3, the plasmid curing efficiency was the same for strains with different numbers of integrations, and the plasmid elimination efficiency reached the highest value of 98% at an induction time of 3 h. The plasmid elimination efficiency of *E. coli* with the pCas protein in the medium was 98%.

**FIGURE 4:**
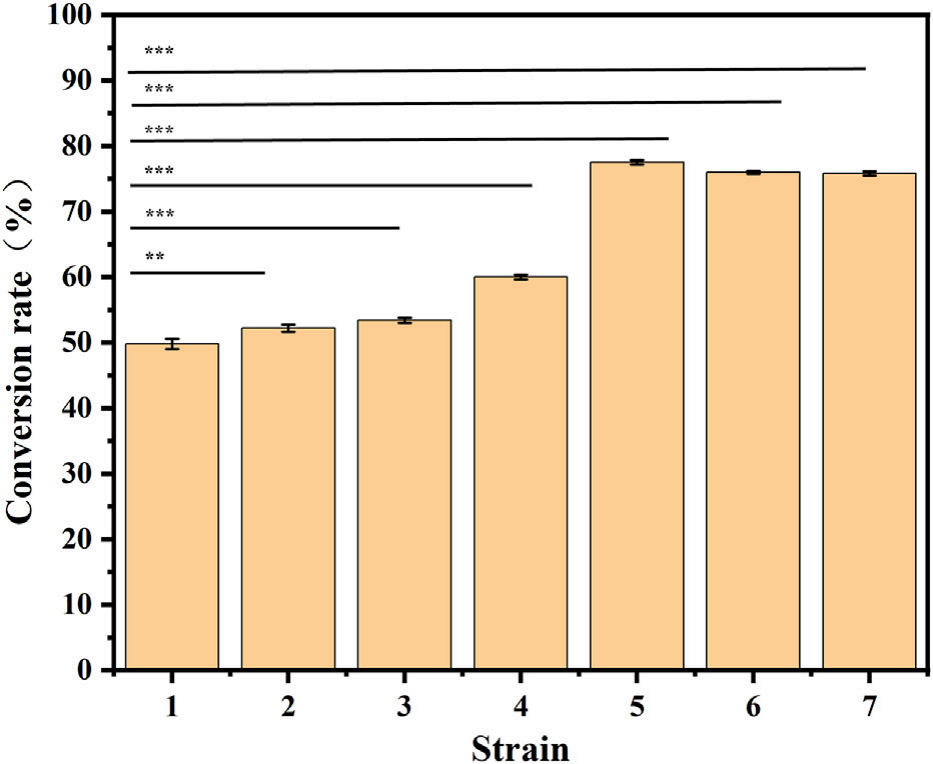
Substrate conversion rates for whole-cell catalyzed synthesis of 10-HDA using decanoic acid as substrate in 1-7 generation *mexHID* genome-integrated strains. Error bars represent standard deviations.

**FIGURE 5:**
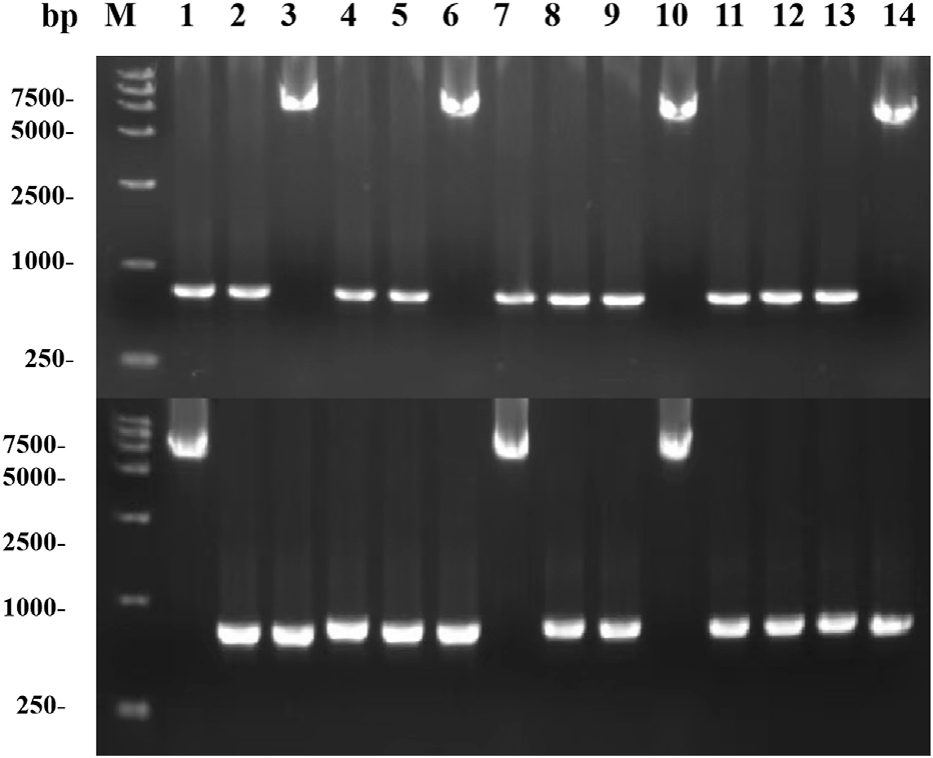
Copy number validation based on PCR of strain E. coli-AKS-mexHID-5 IS1 locus.

**FIGURE 6:**
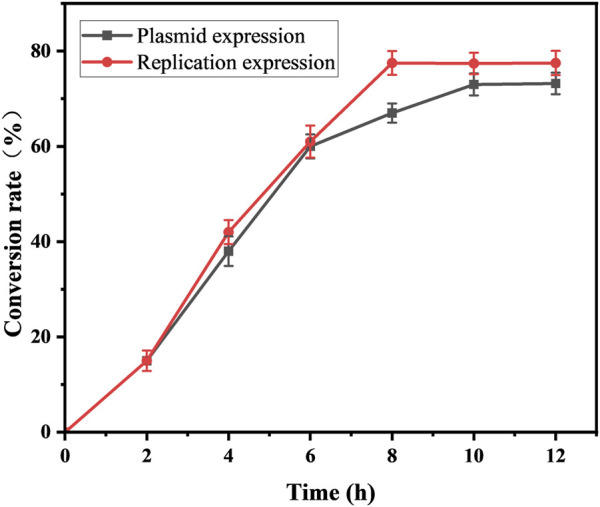
Substrate conversion rates of *mexHID* genome-integrated expression strains and plasmid-expressing strains for whole-cell catalyzed synthesis of 10-HDA using decanoic acid as substrate. Error bars represent standard deviations.

With an increase in the number of induced passages, the integration number of the *mexHID* gene in the strain genome will increase accordingly, and the mRNA abundance of the *mexHID* gene can be analyzed by measuring the mRNA abundance of the *mexHID* gene by qPCR reaction (Peng et al., 2023). The mRNA abundance of the *mexHID* gene in *E. coli*-AKS-mexHID-5 was 6.6-fold higher (p < 0.001) than that in *E. coli*-AKS-*mexHID*-1, indicating that the *mexHID* gene in *E. coli*/E5 possessed higher expression. In generations 1–7, the strains underwent multiple rounds of induced passages, resulting in more *mexHID* genes being integrated into the IS position in the strain genome, and thus, the mRNA abundance of the *mexHID* gene gradually increased and reached a maximum copy number of seven in the fifth generation of strains (Supplementary Figure S4). Whole-cell catalytic assays were performed with generations 1–7 of bacteria bearing genomic copies of the *mexHID* genes (Figure 4) The number of passages was found to be positively correlated with copy number and product conversion in generations 1-5, and the transformation rate of the fifth generation strain, *E. coli-*AKS-*mexHID*, could reach a maximum of 77.5%. To further validate the copy number we extracted the genome of *E. coli*-AKS-*mexHID*-5 and performed primer design and pcr validation of 28 IS1 loci, the primer list is shown in TableS1, the gel electrophoresis graph is shown in Figure 5, Specific bands appeared at seven loci, confirming the copy number of 7. Comparison of the catalytic efficiency of the fifth generation strain *E. coli*-AKS-*mexHID*-5 with that of the strain *E. coli*-AKS-*mexHID* (plasmid expression) expressing the transporter protein in plasmid form revealed that copying the mexHID gene to the genome was more stable and had a higher conversion rate of 4% than the expression of the free plasmid (Figure 6).

### 3.3 Whole-cell catalyzed synthesis of 10-HDA using recombinant strains

**FIGURE 7:**
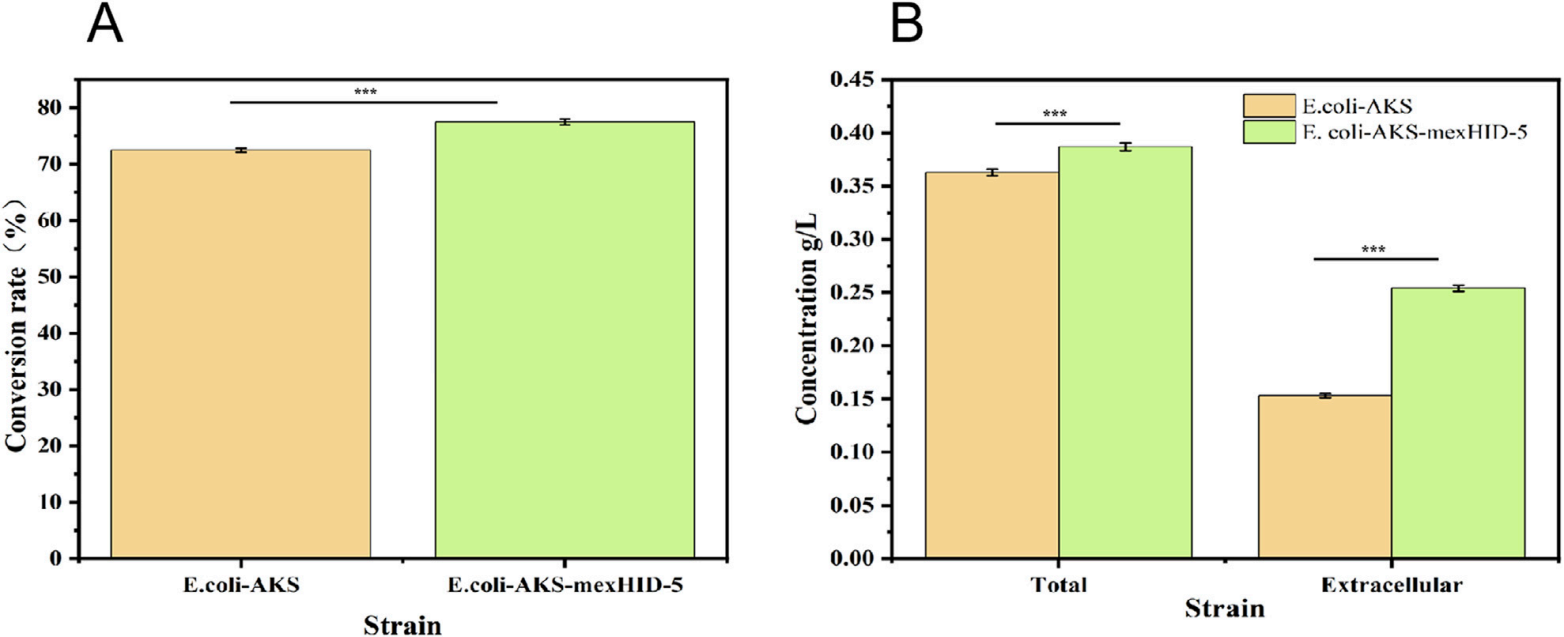
**(A)** Comparison of substrate conversion rates for whole-cell catalyzed synthesis of 10-HDA by *Escherichia coli*-AKS-*mexHID*-5 genome-integrated strains and *Escherichia coli*-AKS strains. Error bars represent standard deviations. **(B)** Comparison of intra- and extracellular 10-HDA content during whole-cell catalyzed synthesis of 10-HDA in *Escherichia coli*-AKS-*mexHID*-5 genome-integrated strains and *Escherichia coli*-AKS strains. Error bars represent standard deviations.

**FIGURE 8:**
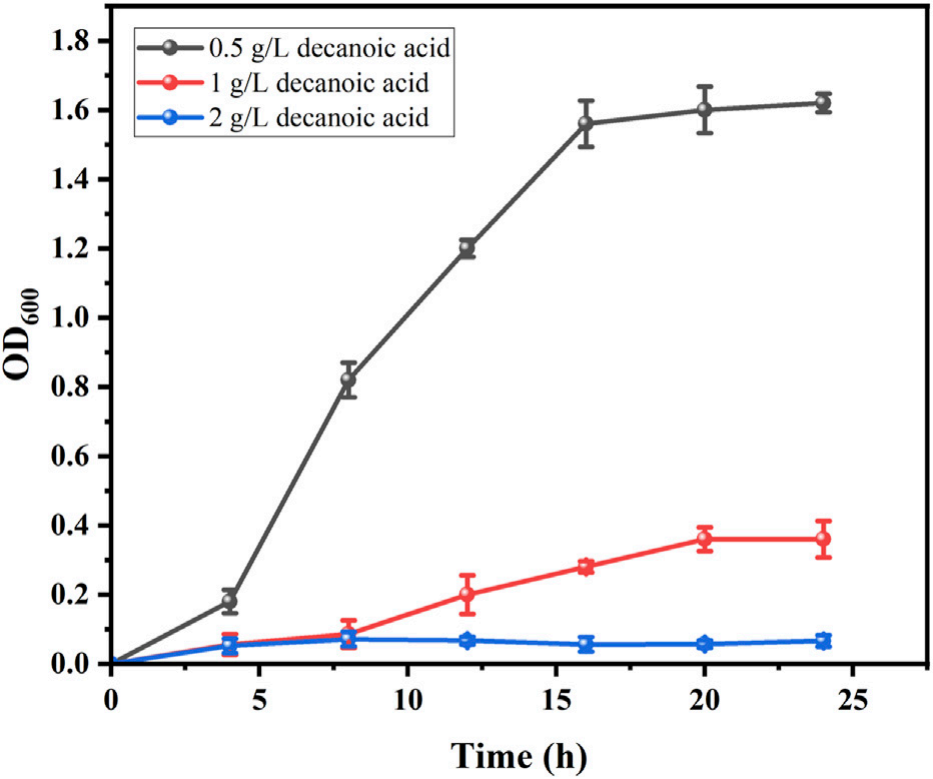
Growth curves of *E. coli*-AKS-*mexHID*-5 at different decanoic acid concentrations. Error bars represent standard deviations.

**FIGURE 9:**
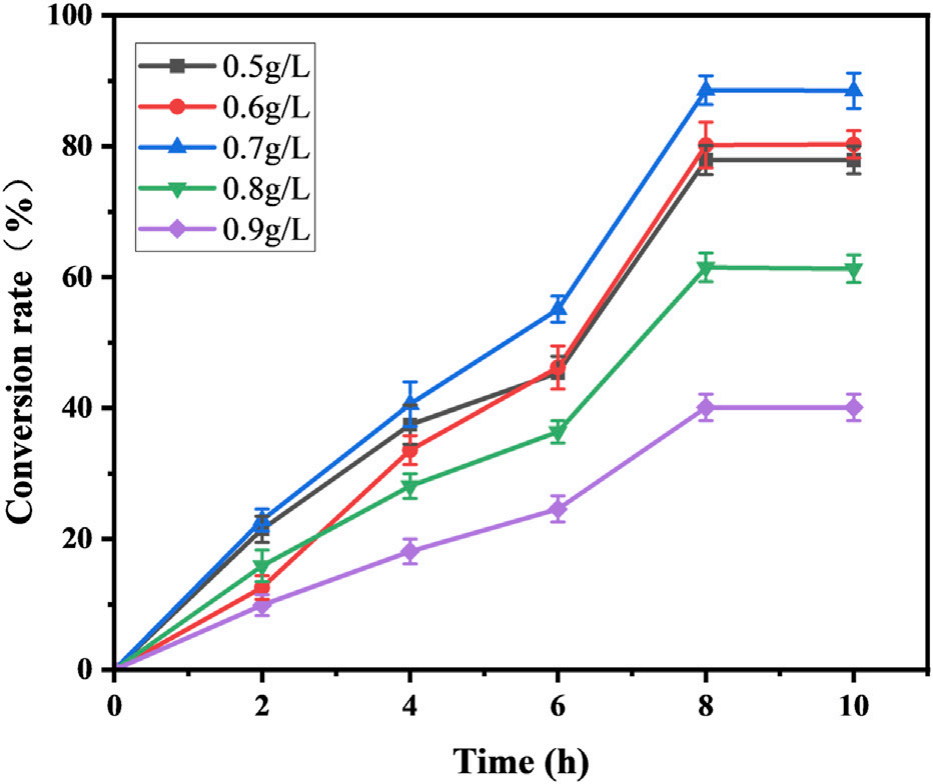
Substrate conversion rates for whole-cell catalyzed synthesis of 10-HDA by *Escherichia coli*-AKS-*mexHID*-5 strains at different decanoic acid addition concentrations. Error bars represent standard deviations.

**FIGURE 10:**
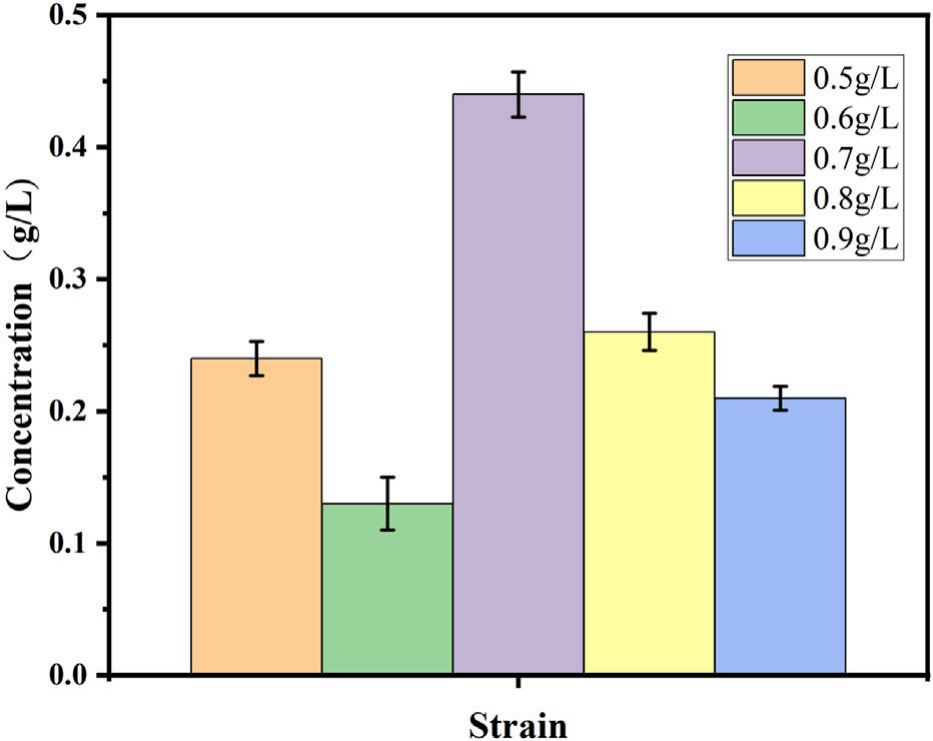
Extracellular 10-HDA content during whole-cell catalyzed synthesis of 10-HDA by *Escherichia coli*-AKS-*mexHID*-5 strains at different decanoic acid addition concentrations. Error bars represent standard deviations.

Both decanoic acid and 10-HDA contain 10 carbon atoms and do not require modification of their length during synthesis. In addition, it is cheaper, making it a common substrate for 10-HDA biosynthesis. The strains *E. coli*-AKS-*mexHID*-5 and *E. coli*-AKS were first compared at a substrate concentration of 0.5 g/L decanoic acid. The comparison of the conversion rate is shown in Figure 7A. The strain E. coli-AKS-mexHID-5, which was integrated by multicopy chromosomes, was found to have a 5% higher transformation rate than the original strain *E. coli*-AKS. Total intracellular and extracellular yields were compared with the extracellular yields in Figure 7B. The extracellular yield of *E. coli*-AKS-*mexHID*-5 was found to be 0.1 g/L more than that of *E. coli*-AKS at a substrate concentration of 0.5 g/L. In order to test the optimal initial substrate concentration of the strain, we performed decanoic acid tolerance experiments on *E. coli*-AKS-*mexHID*-5 by observing its growth curves at decanoic acid concentrations of 0.5, 1, and 1.5 g/L (Figure 8), respectively According to the graph, we set the decanoic acid concentration in a concentration gradient between 0.5-1 for the comparison of the conversion rates. We compared the conversion rates of the strains at 500, 600, 700, 800 and 900 mg/L decanoic acid concentrations. co-expression of YdiI, Macs, FadE, CYP, ACOX, and MexHID resulted in 10-HDA synthesis, and after 8 h of reaction, the highest conversion rate of 88.6% was achieved at a substrate concentration of 0.7 g/L (Figure 9). By examining the extracellular 10-HDA yield at different substrate concentrations, it was found that the highest extracellular yield of 0.44 g/L was achieved at a substrate concentration of 0.7 g/L. (Figure 10). It was more validated that 0.7 g/L was the optimal substrate concentration., so 0.7 g/L was the best initial substrate concentration. Four main substances were assayed in the cellular reaction system and their concentrations (decanoic acid, trans-2-decenoic acid, 10-hydroxydecanoic acid, 10-HDA) were monitored at different time points and it was found that after the reaction was completed the percentages of the four substances were as shown in Supplementary Figure S5.

**FIGURE 11:**
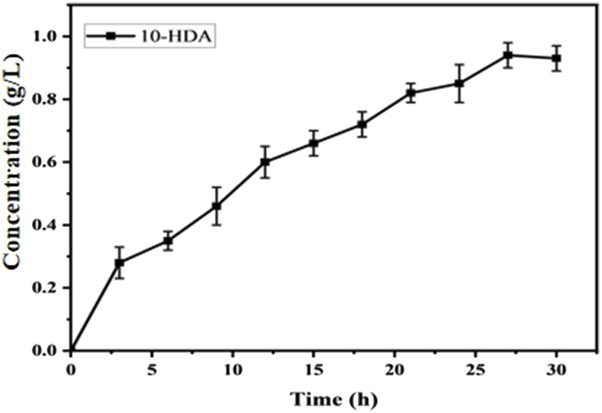
Efficiency of 10-HDA synthesis in *Escherichia coli*-AKS-*mexHID*-5 strains in the substrate plus state. Error bars represent standard deviations.

To further increase the yield of 10-HDA, we used the flow-addition culture technique to optimize the production process of 10-HDA. Flow-addition culture is a semicontinuous culture in which fresh medium is replenished intermittently or continuously to maintain high-density cell growth and increase the yield of the target product (Sriphochanart and Skolpap, 2022; Yu et al., 2023). In a study by Zeng et al., optimization of the fermentation process by replenishment of batch culture resulted in the production of 1,3-dihydroxyacetone (DHA) with a yield of 198.81 g/L in a 5 L bioreactor with a glycerol conversion of 82.84% (Zeng et al., 2022). A flow-through addition experiment was conducted starting at hour eight of the reaction, with 0.1 g/L added every 4 h until the final concentration of 1.3 g/L was reached, at which time the reaction was completed. The highest 10-HDA concentration of 0.94 g/L was reached when the concentration of decanoic acid in the flow-addition reached 1.2 g/L (Figure 11). We found that the yield could be significantly increased by transporting 10-HDA from intracellular to extracellular space using a suitable transporter protein. This strategy not only reduced the intracellular accumulation of 10-HDA and decreased its toxicity to cells but also increased the yield. This provides an efficient and economical method for the industrial production of 10-HDA. By optimizing the substrate addition strategy and improving the utilization rate of the substrate, our method is simple, provides high product purity, and is suitable for industrial production. By modulating the substrate addition strategy and utilizing transporter proteins, we successfully optimized the production of 10-HDA and achieved a significant increase in yield. This study provides important technical support for the industrial production of 10-HDA.

## Data Availability

The original contributions presented in the study are included in the article/Supplementary Material, further inquiries can be directed to the corresponding author.

## Abbreviations

10-HDA, 10-hydroxy-2-decenoic acid; PBS, phosphate-buffered saline; GC-MS, gas chromatography-mass spectrometry; IPTG, isopropyl β-D-thiogalactopyranoside; SDS-PAGE, sodium dodecyl sulfate-polyacrylamide gel electrophoresis; PCR, polymerase chain reaction.
