# Supplementary material for: Enhancing 10-HDA production of Escherichia coli by heterologous expression of MexHID transporter proteins
Source: Front Bioeng Biotechnol. 2025 Jun 9;13:1590291. doi: 10.3389/fbioe.2025.1590291 (PMC12183247; doi:10.3389/fbioe.2025.1590291)
Supplement: Supplementary file 1 [file Table1.docx]

**Table S1.**Primers for the IS1 locus

| Primers | Sequence（5’-3’）—F | Sequence（5’-3’）—R |
| --- | --- | --- |
| 1 | agcattcttgatatatcttaatctcttc | atctcttcagaaagacattcg |
| 2 | catttcatgccatttttaatatagattg | gggagtgtgcattctaattt |
| 3 | gataggtcatgccaaccgcgacaa | ccgcgacaatatcatcattattaa |
| 4 | tccggcaggtaaattacgttcta | gtttttttgctaagaataaaatc |
| 5 | cgcatccggcattaaaggaaaatc | aggaaaatcagcaattaacgttgtg |
| 6 | aagtagtggagaaaccgaaacc | accgaaaccatcaccattgg |
| 7 | cggaaagaccagtgatgaacatg | agcgaactgaaggatgctgt |
| 8 | tttttttgcccatttgtcgggc | ttccccataatgatattaagcgtt |
| 9 | atgttgttcaatgttgcaaactga | attaatatctatgtattttgat |
| 10 | gcgggctgattttcaacggca | tcaacggcaaaccgattaccggta |
| 11 | cgtttcttgcctcaaggcgac | aaggcgactatcattatcaaattc |
| 12 | acgtaacgtactgaaaacgg | tgaaaacgggtgaacaatattta |
| 13 | gatgcctcaccgacgacttt | ctcgactggtaaccatccg |
| 14 | gaagaagtctaacaaatatcc | caaatatccttgtgataata |
| 15 | gcgctggcgagctgaaatcg | tgaaatcgctctttaaaccc |
| 16 | ggctcgttttatcccaactac | ctgaccacaatgctgttgag |
| 17 | gagaaatagtgcaggccgtc | gtccggcagataaagactaatcac |
| 18 | ctggttccgatcatcaggttgta | caggttgtaaacgagctc |
| 19 | aaactatttgacccattctcc | cattctccctctctgctg |
| 20 | tttttctttggtctacgtatgc | tagtttactaacggtcattttg |
| 21 | aacaaagttgccttacgatttttg | cgatttttgagagccagcgc |
| 22 | aaaagatattttattcgcttcctg | cgcttcctgaagtaattttt |
| 23 | cgcaacgttgccagcaca | gccagcacatctgccagcac |
| 24 | gtccattcatttcgggaatgttg | gaatgttgaggcgttttg |
| 25 | aatatatagtccctctcgtt | ggcgtgttgttgcaacatc |
| 26 | gaatatgggaatagatacgcg | gatacgcgacttatccagga |
| 27 | agagcgaattcataatgatggata | acggcttttaaaggcttaaccc |
| 28 | tattgataatgttattcaaaattg | caaaattgtttcactccagtct |
