# Supplementary figures and images for: Enhancing 10-HDA production of Escherichia coli by heterologous expression of MexHID transporter proteins
Source: Front Bioeng Biotechnol. 2025 Jun 9;13:1590291. doi: 10.3389/fbioe.2025.1590291 (PMC12183247; doi:10.3389/fbioe.2025.1590291)

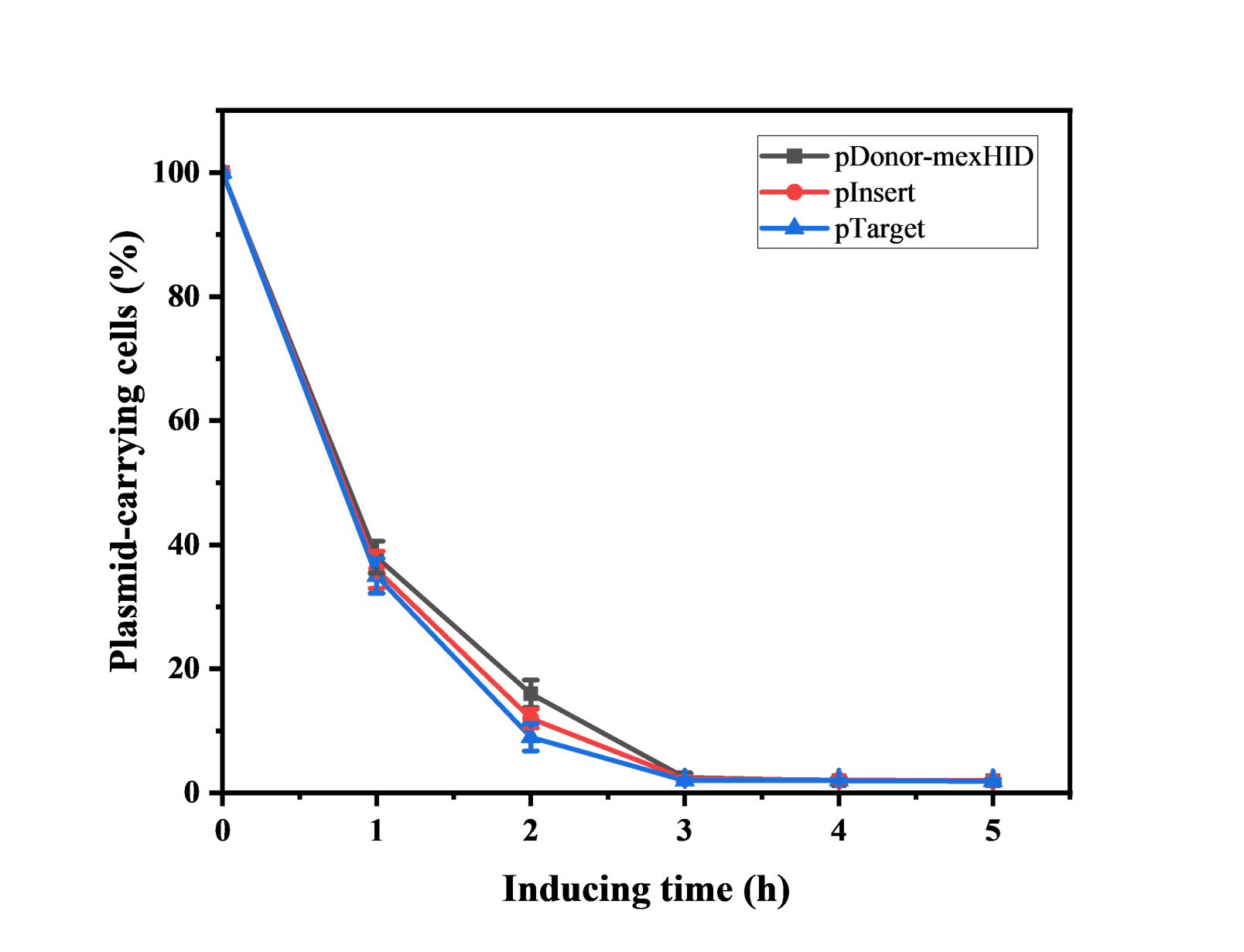

Supplement: Supplementary file 2 [file Image3.tif]

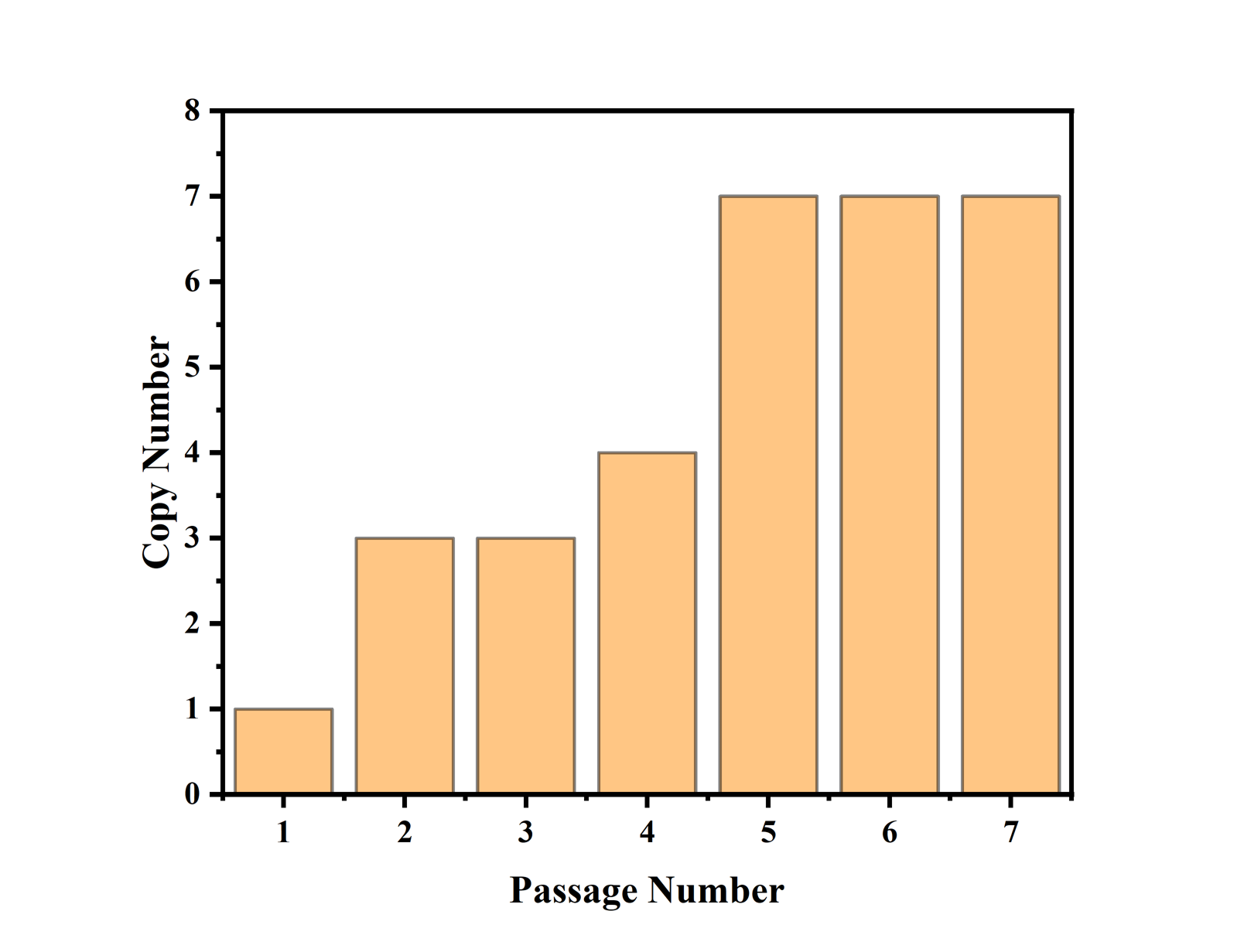

Supplement: Supplementary file 3 [file Image4.tif]

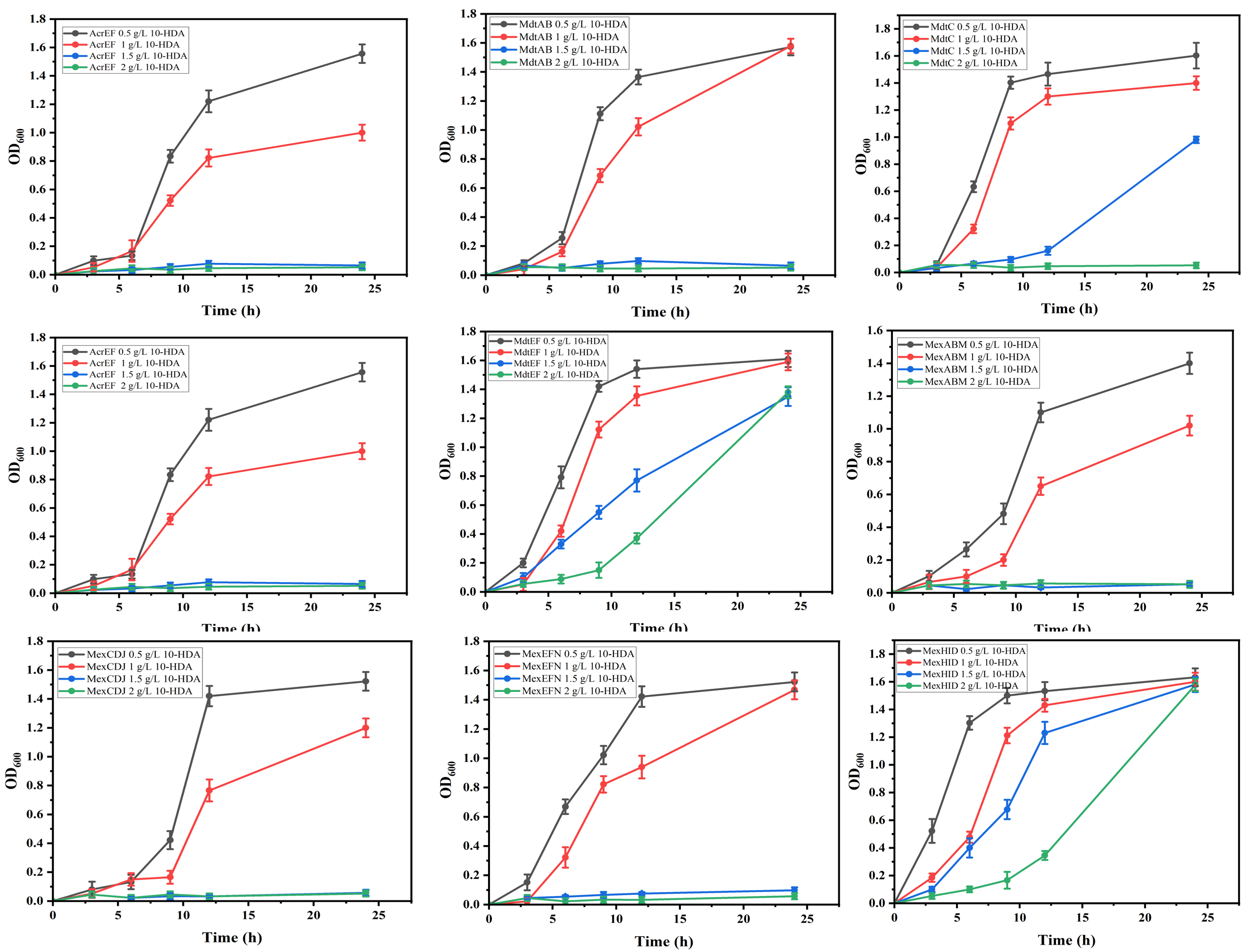

Supplement: Supplementary file 4 [file Image2.tif]

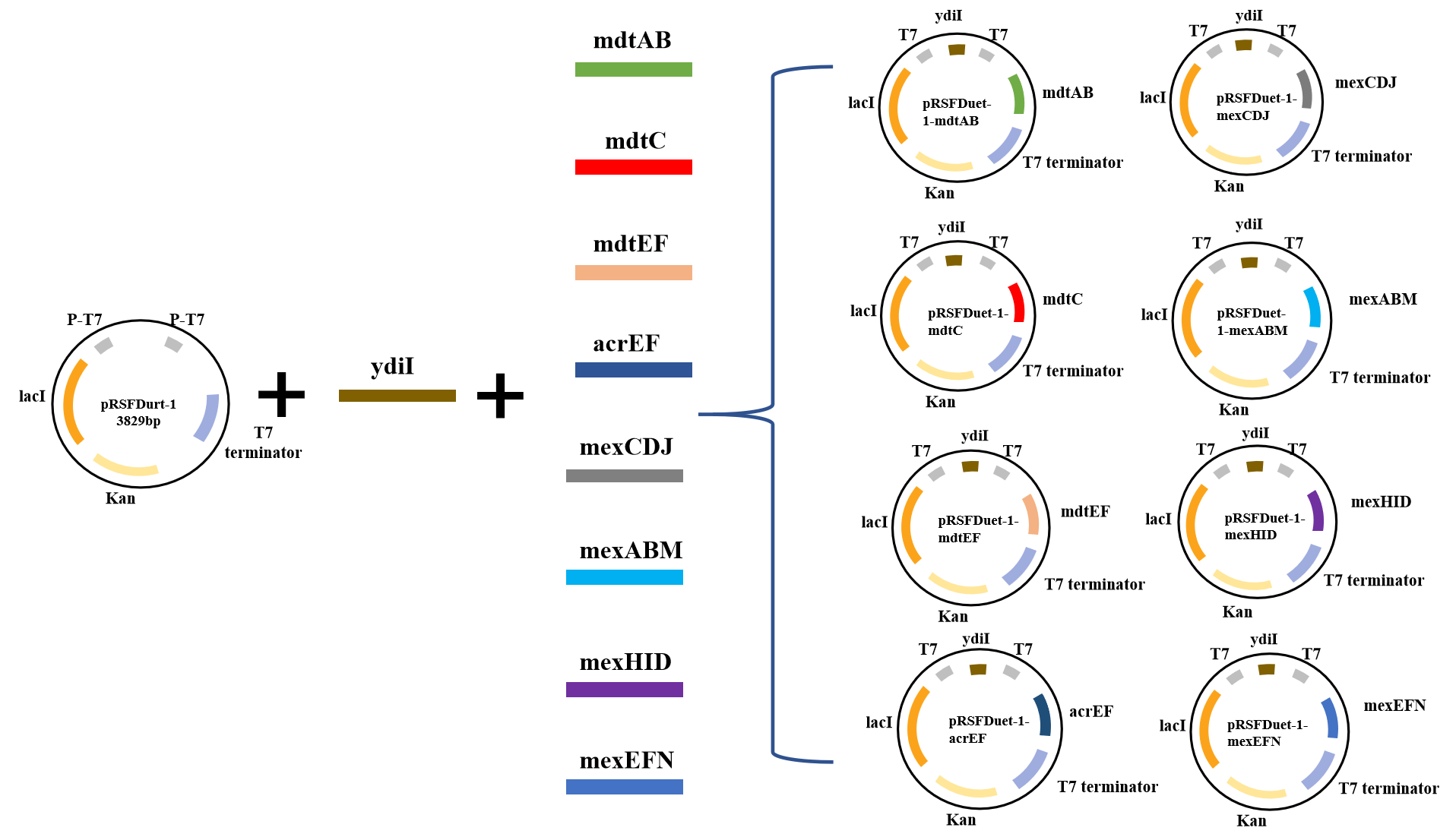

Supplement: Supplementary file 5 [file Image1.tif]

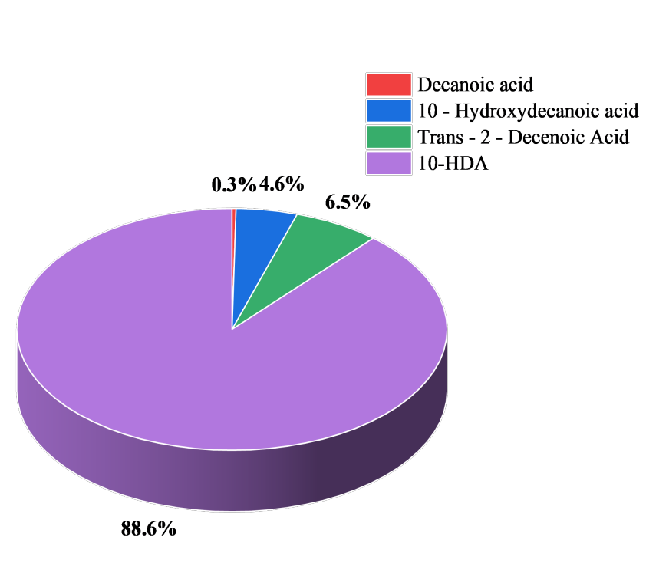

Supplement: Supplementary file 6 [file Image5.tif]
